# The Impact of an Adolescent-Friendly Approach on Metabolic Control in Adolescents with Type 1 Diabetes in Türkiye

**DOI:** 10.3390/jcm14207377

**Published:** 2025-10-18

**Authors:** Ayşegül Şahiner, Ayşe Gül Güven, Zeynep Şıklar, Merih Berberoğlu, Elif Özsu, Zehra Aycan

**Affiliations:** 1 Division of Adolescent Health, Department of Pediatrics, Ankara University School of Medicine, Ankara 06590, Türkiye; aysegulguven1@gmail.com (A.G.G.);; 2 Department of Adolescent Health, Institute of Health Sciences, Ankara University, Ankara 06590, Türkiye; 3 Division of Pediatric Endocrinology, Department of Pediatrics, Ankara University School of Medicine, Ankara 06590, Türkiye

**Keywords:** adolescent, metabolic control, type 1 diabetes

## Abstract

**Background/Objectives**: Metabolic control worsens in adolescents with Type 1 Diabetes. In this study, adolescent-friendly structured adolescent interviews were conducted to identify psychosocial problems. The effect of providing the necessary support for these problems on metabolic control was evaluated. **Methods**: In this study, a survey asking about personal, family, school, and diabetes-related information was administered face-to-face to 21 adolescents with Type 1 diabetes and poor metabolic control. At each visit, the structured adolescent interview was conducted under the headings of HEEADSSS (home/education/eating/activity/addiction/depression/safety). Patients who required it were seen by the adolescent health department’s dietitian and psychologist using a transdisciplinary approach. The patients’ HbA1c levels were compared before and after the study, with a minimum follow-up period of 6 months. **Results**: Of the 21 patients with a calendar age of 13.6 ± 1.7 years and a diabetes duration of 6.4 ± 3.6 years, 14 (66.7%) were female. The mean pre-study HbA1c level was 10.9 ± 1.9%, while the post-study level was 9.3 ± 1.9% (*p* = 0.002). While 100% of patients had poor metabolic control before the study, this rate decreased to 47.6% with the adolescent-friendly approach. No significant difference was found between the two groups (those with improved metabolic control and those without) in terms of relationship with mother, relationship with father, family relationships, mother and father education level, school success, and screen time. **Conclusions**: The adolescent-friendly approach has a highly positive effect on the metabolic control of adolescents with Type 1 diabetes, and this approach should be adopted by physicians who follow this patient group.

## 1. Introduction

Type 1 diabetes (T1D) is one of the chronic childhood diseases that is difficult to manage. A total of 9.5 million people living with clinically diagnosed T1D globally in 2025. Of these, 1.9 million (19.0%) are <20 years (with 1.0 million aged <15 years). Türkiye ranks 8th among the countries with the highest number of people with T1D aged <20 years [1]. Due to the absolute insulin deficiency in T1D insulin replacement (via injections or continuous infusion systems), frequent blood glucose monitoring, regimented diet, and structured exercise programs are required [2]. Although recent technological advances (such as continuous glucose monitoring systems and insulin pumps) have significantly improved metabolic control and quality of life, optimal metabolic control in T1D has not yet been achieved globally or in Türkiye [3]. Achieving target HbA1c levels is particularly challenging during adolescence. Improving metabolic control and maintaining near-normal blood glucose levels are essential in preventing microvascular and macrovascular complications of T1D [4]. A 1% reduction in HbA1c has been shown to reduce the risk of retinopathy by 35%, nephropathy by 24–44%, and neuropathy by 30% [5]. Adolescence is a turbulent period of transition from childhood to adulthood characterized by various biophysical, social, and cognitive changes. During this time, efforts towards individualization and distancing from the family often lead to reduced adherence to treatment for chronic diseases. Similarly, interruptions in insulin therapy, poor dietary compliance, and lack of glucose monitoring can negatively affect metabolic control in adolescents with T1D [4]. Studies have shown that over two-thirds of adolescents with T1D have HbA1c levels above 7.5% [6]. In addition to the physiological insulin resistance caused by body composition and hormonal changes during puberty, factors such as family conflicts, peer relationships, academic difficulties, and the adolescents’ desire for autonomy can also negatively impact metabolic control [4,7,8].

The Society for Adolescent Health and Medicine (SAHM) emphasizes the importance of providing confidential consultations and psychosocial assessments in settings without parents. The HEEADSSS approach is recommended for evaluation by SAHM. HEEADSSS is an acronym for Home, Education, Eating, Activities, Drugs, Sexuality, Safety, and Suicide, which is designed to help healthcare professionals assess adolescents’ psychosocial environments [7]. The International Society for Pediatric and Adolescent Diabetes (ISPAD) recommends recognizing adolescents’ need for privacy and confidentiality while maintaining parental trust and support, and fostering self-confidence and self-efficacy [3]. When adolescents are assured of confidentiality, they are more likely to seek healthcare, disclose sensitive issues, and return for future visits [7]. The qualities of the medical professional play a crucial role in establishing a relaxing atmosphere for the adolescent. In this respect, the American Diabetes Association (ADA) recommends that a multidisciplinary team trained in pediatric diabetes care provide adolescent-specific care [9].

Self-efficacy refers to the belief in one’s ability to perform specific behaviors in certain situations. Self-efficacy is strongly associated with adherence to diabetes self-management among adolescents [10]. Self-management is the most recognized individual factor influencing glycemic control. Studies have shown a direct correlation between better self-management and lower HbA1c levels in adolescents with T1D [9]. Cross-sectional studies have found that high HbA1c levels are associated with poorer self-management [11]. In addition to reduced insulin sensitivity during adolescence, factors such as psychosocial changes, lack of school support, changing eating patterns, and increased snacking may further complicate T1D management [10].

The demands of planning and constant self-monitoring often conflict with adolescents’ desire for immediate gratification, especially in the presence of peers. Given this developmental context, adolescents with T1D need to be equipped with regulatory skills to resist temptations and maintain self-control when faced with psychosocial stressors [12,13]. Effective interventions for children and adolescents with T1D often aim for improved coping and problem-solving skills, resulting in a better quality of life. Interventions that focus on enhancing parent–child collaboration have been shown to positively affect T1D control [7].

According to the World Health Organization (WHO) guidelines, for healthcare services to be adolescent-friendly, they must demonstrate an approach that understands adolescents, respects their privacy and individualization, and includes them in decision-making processes. Furthermore, health service providers are non-judgmental and considerate in their dealings with adolescents, and they have the competencies needed to deliver the right health services in the right way [14]. The current study aimed to examine the impact of an “adolescent-friendly approach on metabolic control in adolescents with T1D. The adolescent-friendly approach respects adolescent individuality, promotes collaboration and inclusion in decision making, and strengthens self-efficacy.

## 2. Materials and Methods

### 2.1. Participant Selection

This study is a clinical intervention based on the findings of a thesis conducted at Ankara University Faculty of Medicine. The thesis revealed that environmental factors such as family, socioeconomic status, and school had no significant effect on metabolic control, whereas diabetes self-management of adolescents played a key role [15].

The current study was conducted on 21 adolescents with T1D. The participants were under the care of the Pediatric Endocrinology Department at Ankara University and were referred to the Adolescent Health outpatient clinic due to poor metabolic control. Good metabolic control was defined as an HbA1c level below 7%, moderate control as 7–9%, and poor control as above 9%. Regardless of their HbA1c level, patients with frequent episodes of hyperglycemia and hypoglycemia were also considered to have poorly controlled T1D [16]. Adolescents with T1D were included in the study based on specific criteria. Accordingly, adolescents who have been under the treatment for a diagnosis of T1D for at least one year, are aged between 10 and 19, and have poor metabolic control (HbA1c ≥ 9% or frequent hyper-/hypoglycemic episodes) were included in the study. Patients with other chronic diseases or psychiatric diagnoses were excluded.

### 2.2. The Survey

The researcher developed a 65-item survey based on the review of the related literature. The survey items focused on personal, family, school, and diabetes-related factors. Since the study was conducted in Türkiye, the survey was prepared in Turkish. The survey was implemented to the participants face to face during their initial visit. Adolescents rated their diabetes management and family relationships on a scale of 1 (poor) to 10 (excellent).

### 2.3. The Adolescent-Friendly Approach

The current study aimed to examine the impact of an “adolescent-friendly approach on metabolic control in adolescents with T1D. This approach included a psychosocial evaluation using the HEEADSSS framework [17]. Besides, consultations with a psychologist and dietitian were organized in the same setting when necessary. The structured interview was conducted by a doctoral student who specialises in adolescent health. Each interview session lasted between 45 and 60 min. The interviews covered topics such as home life, education, eating habits, leisure activities, drug use, sexual behaviour, depression, and safety. The discussions addressed psychosocial issues, deficiencies in T1D management, and strategies for coping with them. A safe and confidential environment was selected for the interviews, enabling adolescents to express themselves freely. The follow-up sessions were built on preceding interviews and were personalized. All interviews were conducted by the same trained doctoral student using motivational interviewing techniques. When necessary, adolescents with T1D were evaluated by a multidisciplinary team including a psychologist and a dietitian. The frequency of visits was tailored to each adolescent and continued for a minimum of six months at intervals of 1–3 months. The number of visits varied from 3 to 13 over a period of 6 to 18 months. This frequency and interval were determined based on the opinions obtained from individual interviews. Adolescents with T1D HbA1c levels were compared before and after the intervention over at least six months of follow-up. HbA1c measurements were performed using Lifotronic H100 Hemoglobin Analyzer (Shenzhen, China) instrument, measured using High-Performance Liquid Chromatography (HPLC) chemiluminescence technique, using Abbott Architect HbA1c kits (4P72) with a measurement range of 2.8–25%.

### 2.4. Ethical Statement

Approval was granted by the Ankara University Faculty of Medicine Ethics Committee on 2 August 2023 (decision no. 2023/411). The purpose of the study was explained to the participants and their families, and informed consent was obtained from all of them.

### 2.5. Analysis

IBM SPSS Statistics version 30.0 was used for data analysis. Categorical data were expressed as frequency and n, and continuous data were expressed as mean ± SD. Chi-square was used for categorical data analysis, and Student’s t-test was used for independent continuous data analysis. A paired sample t-test was used to measure changes in HbA1c. *p* < 0.05 was considered significant.

## 3. Results

Of the 21 adolescents with T1D included in this study, 14 (66.7%) were female, and 7 (33.3%) were male. The mean age was 13.6 years (range: 10–16, SD: 1.77), and the mean duration of T1D was 6.4 years (range: 1–14). Among parents, 19.5% had a university degree, while 7.3% were illiterate. The proportion of adolescents with T1D living with both parents was found to be 90.5%. The mean score for family relationships was 7.8/10; the mother–child relationship score was found as 8.6/10, while the father–child relationship score was 7.4/10. Among the participants, 66.7% (n = 14) had lower academic success. The mean exercise duration was 29.2 min/day (range: 0–120 min/day), and the mean screen time was 3.9 h/day (range: 1–6 h/day). Categorical and continuous sociodemographic data are given in Table 1 and Table 2.

The results of the present study revealed that only 3 adolescents with T1D (14.3%) regularly monitored their blood glucose levels, and 2 adolescents with T1D (9.5%) consistently recorded their glucose values. The mean score of diet compliance was 5.2/10 (range: 3–10). A total of 17 adolescents with T1D (81%) regularly attended diabetes outpatient clinic appointments, while 4 (19%) missed follow-ups. Shared responsibility among mother, father, and child was observed in 9 cases (42.9%). In 3 cases (14.3%), the mother was the sole responsible, and in another 3 cases (14.3%), the adolescent managed their T1D. Regarding carbohydrate counting, 6 adolescents with T1D (28.6%) did not know about it, and 33.3% did not practice it. Among the participants 7 adolescents with T1D (33.3%) used glucose sensors. Only 2 of these adolescents with T1D monitored their glucose levels regularly. The remaining 5 either did not carry the connected phone or ignored the data. As sensors were only included in the national reimbursement list as of December 2024, usage rates were low at the time of this study. Two adolescents with type 1 diabetes (T1D) were found to have HbA1c levels below 9% (8.1%) and were using insulin pumps. It was observed that these two patients experienced frequent episodes of hyperglycemia and hypoglycemia due to overfeeding and corrective insulin boluses. Following intervention with the adolescent-friendly model, these episodes were prevented. Moreover, their HbA1c levels improved to 7.3% and 7.4%, respectively.

**Figure 1:**
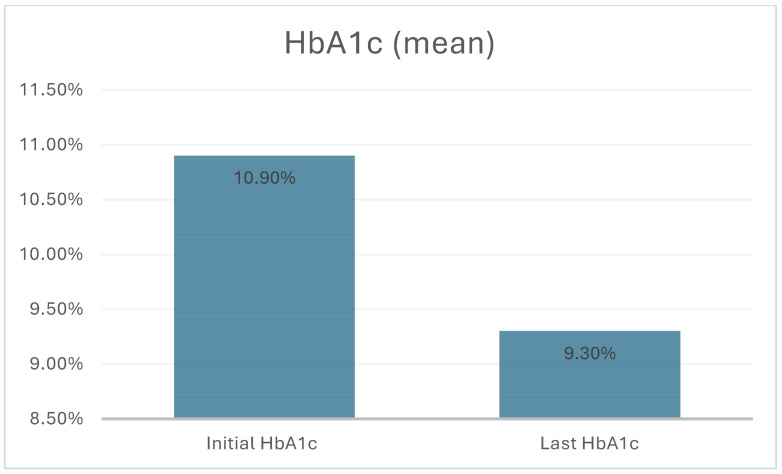
Initial and last HbA1c levels (*p* = 0.001).

The average number of visits to the Adolescent Health Department was 4 (range: 3–13). The mean pre-intervention HbA1c level was 10.9% (range: 8.1–14.6), while the post-intervention mean was 9.3% (range: 7.3–16) (*p* = 0.001) (Figure 1). Additionally, 11 of the 21 adolescents with T1D (52.3%) showed improvement in metabolic control. Among 19 patients with initial HbA1c above 9%, 9 achieved a level below 9%. Notably, the 2 with frequent glycemic episodes despite lower HbA1c experienced resolution of these episodes. The study revealed no significant differences between the two groups, i.e., the improved and non-improved groups, with regard to the following variables: relationships with parents, parental education, school performance, exercise duration, or screen time.

## 4. Discussion

This is the first intervention study designed to examine the hypothesis that strengthening self-management mechanisms can improve metabolic control in adolescents with poorly controlled T1D. In this study, a significant reduction in HbA1c levels was observed in adolescents with T1D who received care based on the adolescent-friendly approach (pre-intervention HbA1c: 10.9%, post-intervention HbA1c: 9.3%) (*p* = 0.001). It is suggested in the related literature that socioeconomic and demographic factors do not significantly influence metabolic control. Instead, personality traits are reported to be important predictors of how well patients adapt to their T1D. Furthermore, inadequacies in self-management mechanisms have been found to be associated with poor blood glucose monitoring and elevated HbA1c levels [6,12,18,19].

It is demonstrated that effective improvement of glycemic control in adolescents with T1D requires a simultaneous focus on individual, peer, and parental factors. It is emphasized that regular assessment of self-management and diabetes-related distress and providing early intervention should be the core strategy to improve glycemic control in adolescents with T1D [6].

Adolescence is characterized by physiological and behavioral changes that affect blood glucose levels. These include pubertal endocrine changes that lead to increased insulin resistance, reduced impulse control reflected in irregular eating and exercise habits, poor adherence to treatment regimens, and involvement in risky behaviors due to poor impulse regulation. In such cases, focusing solely on medical treatment protocols is insufficient for improving glycemic control in adolescents with T1D [3]. In a systematic review published in 2024, it was emphasized that rather than focusing solely on the “diabetological” aspects of the patient, integrated care strategies that approach the patient as a whole should be the most widely recommended approach for managing the disease across all levels of care. The review also demonstrated that school nurse interventions are associated with better adherence to treatment protocols and improved health outcomes [20]. The school nursing system, which is important for improving school health, is not sufficiently supported in Turkey. In order to address the problems faced by children with diabetes at school, “Diabetes at School Program” was launched in November 2010 by the Turkish Society for Pediatric Endocrinology and Diabetes, in collaboration with the Ministry of National Education and the Ministry of Health. Within the scope of this program, numerous activities have been carried out over the past 15 years, leading to nationwide improvements in the care of children with diabetes at school [21].

Another study found that only 13% of adolescents with T1D had participated in adolescent-specific consultations. Practical barriers to individualized adolescent interviews may include time constraints, a lack of provider competence in psychosocial assessment, and a lack of confidence in responding to potential risks that may emerge during such evaluations. In that study, parents reported placing great importance on the support of healthcare professionals in helping their children navigate developmental transitions and prepare for greater responsibilities. The same study also stated that adopting an adolescent-friendly approach to the care of diabetic adolescents is essential and that investigating its impact on glycemic control would be beneficial [7].

A shared conclusion of many studies is that, in adolescents with T1D, responsibility for disease management should shift from the parents to the adolescent. Improving self-management has the potential to enhance metabolic control, and, when necessary, this should be supported by a multidisciplinary care team [7,10,18].

In this study, the adolescent-friendly model implemented in the Department of Adolescent Health included a structured adolescent interview using the HEEADSSS framework, a comprehensive psychosocial assessment, evaluation of all risks that could affect treatment adherence, application of motivational interviewing techniques, and multidisciplinary management involving psychologists and dietitians when needed. This intervention resulted in an average HbA1c reduction of 1.6 percentage points. A 10% decrease in HbA1c (e.g., from 10% to 9%) is associated with a 39% reduction in the risk of retinopathy and a 25% reduction in the risk of microalbuminuria (i.e., neuropathy) [22]. The absence of significant differences between the improved and unimproved groups in terms of maternal and paternal relationships, family dynamics, parental education, academic performance, and screen time supports our view that metabolic control is closely associated with self-management mechanisms.

In our study, the majority of adolescents with poor metabolic control were female. This finding is consistent with several studies in the literature in which female gender was also associated with poorer metabolic outcomes [23,24].

When we examined our findings, we observed that the parents—particularly the mothers—of adolescents with poor metabolic control tended to have a lower level of education. In a study conducted by Nielsen and colleagues, a 41.2% difference in patients’ HbA1c levels was found between the mothers with a high school education and those with a postgraduate degree [25]. Higher education levels of parents have been associated with better self-management and improved glycemic control in children [25,26].

In our study, only 14% of adolescents with T1D measured their blood glucose regularly, and only 9.5% kept records. The vast majority of adolescents with T1D did not practice carbohydrate counting. Similar findings have been reported in the literature, emphasizing that adolescents with poor self-regulation during puberty often struggle with glucose monitoring, record keeping, accurate insulin dosing, and administration—factors that collectively contribute to poor metabolic control [23,25,26].

Additionally, adolescents with T1D were found to have long screen time and poor academic performance. These findings suggest that deficiencies in self-management mechanisms may extend beyond diabetes care to other aspects of their lives.

## 5. Limitations

This study has several limitations. Since this was the first intervention study on this subject, Q-power analysis could not be performed. The number of adolescents with T1D was small, and no control group was included. In the study design, it was considered that an ethical problem might arise for untreated patients with poorly controlled diabetes; therefore, it was deemed more appropriate to compare pre- and post-HbA1c values. Poor metabolic control was defined as HbA1c ≥ 9% or frequent hyper-/hypoglycemic episodes, according to the Turkish Pediatric Endocrinology and Diabetes Society’s guidelines for the diagnosis and treatment of T1D, and the study was conducted in a single center, which limited the number of participants. According to the ISPAD Clinical Practice Consensus Guidelines 2022, poor control is defined as HbA1c > 7%. Therefore, multicenter studies involving a larger number of adolescents with T1D and HbA1c levels above 7% are needed.

## 6. Conclusions

This study found that the adolescent-friendly approach in adolescents with T1D was associated with increased treatment adherence and a reduction in HbA1c levels, indicating improvements in metabolic control. Although programs such as “school nursing” and “diabetes at school programs” are utilized for this purpose, challenges in managing T1D during adolescence still persist. In the management of chronic diseases such as T1D, especially during adolescence, the application of medical treatment protocols alone may be insufficient. The development of multidisciplinary interventions grounded in adolescent-friendly approaches can enhance treatment compliance in these patients.

Departments of Adolescent Health can play a crucial role in the management of chronic illnesses such as T1D. They can adopt an approach that not only acknowledges the physical, social, and psychological changes in adolescents but also respects their need for privacy and confidentiality. At the same time, this approach supports adolescents’ self-confidence and self-efficacy while maintaining the trust and support of parents.

## Figures and Tables

**Table 1:** Categorical sociodemographic data.

Variable	Categories	N	Percent (%)
Gender	Girl	14	66.7
	Boy	7	33.3
Carbohydrate Counting	Knows & applies	8	38.1
	Knows but doesn’t apply	7	33.3
	Doesn’t know	6	28.6
Both Parents Live Together	Yes	19	90.5
	No	2	9.5
Academic Success	Good	2	9.5
	Moderate	5	23.8
	Poor	14	66.7
Mother’s Education	Illiterate	2	9.5
	Primary School	10	46.7
	High School	6	28.6
	University	3	14.3
Father’s Education	Primary School		
	High School	5	23.8
	University	11	52.4
		5	23.8

**Table 2:** Continuous sociodemographic data. ^a^ A scale of 1 (poor) to 10 (excellent).

Variable	Minimum	Maximum	Mean	Std. Deviation
Age	10	16	13.67	1.77
Duration of Diabetes (years) ^a^	1	14	6.43	3.70
Score of Relationship with Mother ^a^	5	10	8.62	1.60
Score of Relationship with Father ^a^	0	10	7.43	3.09
Family Relationship Score ^a^	3	10	7.81	2.16
Diet Compliance Score ^a^	3	10	5.24	1.70
Exercise Duration (minutes)	0	120	29.29	35.96
Screen Time (hours)	1	6	3.90	1.51

## Data Availability

Data available on request due to patient privacy.

## Abbreviations

The following abbreviations are used in this manuscript:

T1DM	Type 1 Diabetes Mellitus
SAHM	The Society for Adolescent Health and Medicine
HEEADSSS	Home, Education, Eating, Activities, Drugs, Sexuality, Safety, Suicide
ISPAD	The International Society for Pediatric and Adolescent Diabetes
ADA	The American Diabetes Association

## References

[1] Ogle G.D., Wang F., Haynes A., Gregory G.A., King T.W., Deng K., Dabelea D., James S., Jenkins A.J., Li X. (2025). Global type 1 diabetes prevalence, incidence, and mortality estimates 2025: Results from the International diabetes Federation Atlas, and the T1D Index Version 3.0. Diabetes Res. Clin. Pract..

[2] Libman I., Haynes A., Lyons S., Pradeep P., Rwagasor E., Tung J.Y.l., Jefferies C.A., Oram R.A., Dabelea D., Craig M.E. (2022). ISPAD Clinical Practice Consensus Guidelines 2022: Definition, epidemiology, and classification of diabetes in children and adolescents. Pediatr. Diabetes.

[3] Gregory J.W., Cameron F.J., Joshi K., Eiswirth M., Garrett C., Garvey K., Agarwal S., Codner E. (2022). ISPAD clinical practice consensus guidelines 2022: Diabetes in adolescence. Pediatr. Diabetes.

[4] Aydın A.İ., Öztaş G., Atak M., Özyazıcıoğlu N., Sağlam H. (2025). The effect of social support and parental monitoring on glycaemic control in adolescents with type 1 diabetes mellitus. J. Eval. Clin. Pract..

[5] Grubu T.D.B.Ç. (2024). DİABETES MELLİTUS VE KOMPLİKASYONLARININ TANI, TEDAVİ VE İZLEM KILAVUZU. https://file.temd.org.tr/Uploads/publications/guides/documents/diabetesmellitus2024.pdf.

[6] Lee S.L., Tsai M.C., Chang S.C., Chen J.L., Wang R.H. (2020). Modelling individual, parental and peer factors to glycaemic control in adolescents with type 1 diabetes: A prospective study. J. Adv. Nurs..

[7] Duncan R.E., Jekel M., O’Connell M.A., Sanci L.A., Sawyer S.M. (2014). Balancing parental involvement with adolescent friendly health care in teenagers with diabetes: Are we getting it right?. J. Adolesc. Health.

[8] Renaud-Charest O., Mok E., Frei J., Brunet M.L., Henderson M., Rahme E., Dasgupta K., Nakhla M. (2024). Diabetes duration, perceived comfort with self-management and glycaemic control in adolescents with type 1 diabetes: A cross-sectional study. Diabet. Med..

[9] Care D. (2023). Standards of care in diabetes—2023. Diabetes Care.

[10] Wibaek R., Ibfelt E.H., Andersen G.S., Hulman A., Dabelea D., Jørgensen M.E., Svensson J., Vistisen D., Rønn P.F. (2024). Heterogeneity in glycaemic control in children and adolescents with type 1 diabetes: A latent class trajectory analysis of Danish nationwide data. Diabet. Med..

[11] Radcliff Z., Weaver P., Chen R., Streisand R., Holmes C. (2018). The role of authoritative parenting in adolescent type 1 diabetes management. J. Pediatr. Psychol..

[12] Rassart J., Oris L., Prikken S., Weets I., Moons P., Luyckx K. (2018). Personality functioning in adolescents and emerging adults with type 1 diabetes. J. Adolesc. Health.

[13] Silva K., Miller V.A. (2019). The role of cognitive and psychosocial maturity in type 1 diabetes management. J. Adolesc. Health.

[14] World Health Organization (WHO) (2012). Making health services adolescent friendly. Developing National Quality Standards for Adolescent Friendly Health Services.

[15] Hawwa Z. (2022). Evaluation of the Effects of Social Environments on Metabolic Control in Adolescents with Type 1 Diabetes. Ph.D. Thesis.

[16] https://cocukendokrindiyabet.org/wp-content/uploads/cocukluk-cagi-diyabeti-tani-ve-tedavi-rehberi-2018.pdf.

[17] HEEADSSS 3.0: The Psychosocial Interview for Adolescents Updated for a New Century Fueled by Media. https://www.contemporarypediatrics.com/view/heeadsss-30-psychosocial-interview-adolescents-updated-new-century-fueled-media.

[18] Nefs G., Albright-Pierce M.R., Kanc K., Feinn R., Wagner J. (2020). Diabetes Self-Management Decrements Mediate the Relation of Stressful Life Events and Hemoglobin A1c—Differences by Race/Ethnicity in Adolescents. J. Adolesc. Health.

[19] Hajomer H.A.E., Elkhidir O.A., Mohammed R., Elhassan S., Abdelrahim A., Mohammed Y.I.A. (2025). Investigating the Association Between Family Socioeconomic Profile and Diabetes Control in Children: A Cross-Sectional Study From Sudan. Endocrinol. Diabetes Metab..

[20] Cangelosi G., Mancin S., Morales Palomares S., Pantanetti P., Quinzi E., Debernardi G., Petrelli F. (2024). Impact of school nurse on managing pediatric type 1 diabetes with technological devices support: A systematic review. Diseases.

[21] Hatun Ş., Mutlu G.Y., Gökçe T., Avcı Ö., Yardım N., Aycan Z., Darendeliler F. (2021). Care and support of children with type 1 diabetes at school: The Turkish experience. J. Clin. Res. Pediatr. Endocrinol..

[22] Genuth S., Nathan D., Shamoon H., Duffy H., Engel S., Engel H., Dahms W., Mayer L., Pendegras S., Zegara H. (2001). Beneficial effects of intensive therapy of diabetes during adolescence: Outcomes after the conclusion of the Diabetes Control and Complications Trial (DCCT). J. Pediatr..

[23] Moore J.M., Snell-Bergeon J.K. (2019). Trajectories of hemoglobin A1c and body mass index z-score over four decades among 2 to 18 year olds with type 1 diabetes. Pediatr. Diabetes.

[24] Pironetti R., Saha M.T., Luukkaala T., Keskinen P. (2023). Sociodemographic factors affecting glycaemic control in Finnish paediatric patients with type 1 diabetes. Endocrinol. Diabetes Metab..

[25] Nielsen N.F., Gaulke A., Eriksen T.M., Svensson J., Skipper N. (2019). Socioeconomic inequality in metabolic control among children with type 1 diabetes: A nationwide longitudinal study of 4,079 Danish children. Diabetes Care.

[26] Martin D., Elie C., Dossier C., Godot C., Gagnayre R., Choleau C., Cahané M., Robert J.J., Group A.S. (2017). Diabetes knowledge in adolescents with type 1 diabetes and their parents and glycemic control. Pediatr. Diabetes.

